# *In Vitro* Anti-*Shigella*, Antioxidant Activities, and Oral Acute Toxicity of Organics Extracts from the Root Bark of *Detarium microcarpum* Guill. and Perr.

**DOI:** 10.1155/2024/1330063

**Published:** 2024-09-06

**Authors:** Hama Hamadou Habibou, Mahamane Idi Issa Abdoulahi, Ikhiri Khalid

**Affiliations:** ^1^ Department of Chemistry Faculty of Science and Technology Abdou Moumouni University, BP 10662, Niamey, Niger; ^2^ Department of Organic Chemistry Faculty of Science University of Yaoundé I, P.O. Box 812, Yaoundé, Cameroon

## Abstract

*Detarium microcarpum* (Fabaceae) is a medicinal plant from the traditional pharmacopeia of Niger used against gastrointestinal disorders and dysentery. This study was designed to assess the *in vitro* anti-*shigella*, antioxidant activities, and oral acute toxicity of extract root barks of *Detarium microcarpum*. The crude extracts were prepared by maceration using methanol, ethanol, dichloromethane, water-ethanol (30/70 v/v), and methanol-dichloromethane (1/1 v/v). The anti-*shigella* activity was performed using the microdilution method coupled with the resazurin-based assay. The antioxidant activity was evaluated by the DPPH^·^ (2, 2-diphényl-1-picrylhydrazyl), ABTS 2, 2′-azino-bis (3-ethylbenzothiazoline-6-sulfonic acid), and H_2_O_2_ assays. The oral acute toxicity was assessed following the Organization for Economic Cooperation and Development (OECD) guidelines. The extracts displayed activity against the *Shigella boydii* with Minimum Inhibitory Concentrations (MICs) from 500 to 1000 *μ*g/mL. The methanolic crude extract of *D. microcarpum* shows good antioxidant activity with the radicals DPPH^·^ and ABTS with inhibitory concentration 50 (IC_50_) at 228 and 191 *µ*g/mL, respectively. The lethal dose 50 (LD_50_) of extract was up to 2000 mg/kg of body weight, and no signs of toxicity were observed. These findings supported the use of *Detarium microcarpum* in the traditional treatment of gastrointestinal disorders.

## 1. Introduction

Microorganisms are widespread in the environment, and the majority of them live on land or in water [1]. In addition to its importance in daily human life for food and nonfood needs (personal hygiene and various household tasks), water is also a means of disseminating pathogens that cause waterborne diseases in humans. Access to drinking water is an obstacle to improve people's health, despite the water supply and sanitation programmes that have been put in place. One in three people in the world (i.e., 2.4 billion people) still lives without adequate sanitation facilities, with sub-Saharan Africa the worst affected [2].

This lack is leading people to turn to alternative sources of water of dubious microbiological quality, such as water from lentic environments (wells, springs, and boreholes) and rivers. Microbial infections are diseases caused by the development, in humans or animals, of yeasts, parasite, virus, or bacteria some species of which are pathogenic. These microbial infections, which were once considered commonplace, are now classified as serious infections that can lead to a high rate of mortality and morbidity in immunocompromised patients and diabetics. Antimicrobial chemotherapy has proved highly effective against superficial infections. However, deep infections remain the most difficult to be treated because of the toxicity of systemic antimicrobials and the emergence of resistance to the most commonly used drugs. The microorganisms most frequently responsible of bacterial infections are *E. coli*, and the genera *Shigella*, *Salmonella*, *Yersinia*, and rarely *Vibrio cholerae*, which are only found in some specific areas such as in low-income countries [3].

In Niger, a study carried out on children aged 0–5 hospitalised or consulted at Niamey National Hospital revealed that the germs most frequently involved in childhood gastroenteritis are the bacteria from the genera *Shigella* and *Salmonella* [4]. In view of the problem caused by microorganism resistance to conventional antibiotics, there is a need for constant renewal of active ingredients [5]. Medicinal plants remain the most important sources of molecules used in the composition of pharmaceutical drugs. It therefore makes sense to continue or even intensify research in this field because these plants remain an almost inexhaustible source of biomolecules.

Ikhiri et al., 1984, and the ACCT (*Agence de Coopération Culturelle et Technique*) report presented by Adjanohoum et al., 1989 reported on the use of plants in traditional pharmacopoeia in Niger and listed many species, including *D. microcarpum* belonging to the Fabaceae family, which is widely used in traditional Niger medicine to treat microbial infections and dysentery syndromes [6, 7]. The aim of this study is to evaluate the anti-*shigella* and antioxidant activities of the extracts from the root bark of *Detarium microcarpum*. In order to know the safety of this plant, oral acute toxicity was evaluated.

## 2. Materials and Methods

### 2.1. Plant Materials

*Detarium microcarpum* root barks were freshly collected in March 2018 in Dosso (Niger). The plant collected was identified in comparison with an authentic sample of the herbarium specimens deposited under number 756 in the laboratory of botanic at the Department of Biology (Faculty of Science and Technology of Abdou Moumouni University). The plant material was pretreated and air-dried at room temperature for two weeks. The plant was then pulverized into powder.

### 2.2. Preparation of Extracts

Twenty-five grams (25 g) of fine powder was soaked into 250 mL of each solvent (methanol, ethanol, dichloromethane, water-ethanol (30/70 v/v), and methanol-dichloromethane (1/1 v/v)) and macerated for 48 hours. The set was shaken under magnetic stirring. After filtration, the solvent was evaporated using a rotavapor (Heidolph). The evaporation was done at 40°C under pressure of 175 mbar for the ethanolic extract and 337 for the methanolic extract, as well as 850 mbar for the dichloromethane extract.

### 2.3. *In Vitro* Anti-*Shigella* Activities

#### 2.3.1. Preparation of Stock Solution

*(1) Preparation of Stock Solutions of Extracts and Reference Antibacterials*. Stock solutions of extracts were prepared at 10 mg/mL by dissolving 10 mg of extracts in 1 mL of 10% DMSO (dimethyl sulfoxide) (Sigma Aldrich). Ciprofloxacin (Sigma Aldrich) used as a positive control was prepared under the same conditions at 1 mg/mL by dissolving 1 mg of powder in 1 mL of acidified distilled water (the final acid concentration is of 0.04 N which is not harmful to the bacterial strains).

#### 2.3.2. Bacteria Strains and Growth Conditions

Three strains *Shigella boydii*, *Shigella sonnei*, and *Shigella flexneri*, provided by Bei Resources, and one clinal isolate *Shigella dysenteria* provided by *Centre Pasteur* of Cameroon to the Antimicrobial and Biocontrol Unit, University of Yaoundé 1, Cameroon, were used for the anti-*Shigella* activity. These bacteria were kept at 4°C and revived 24 hrs prior to each assay on Muller Hinton agar (Sigma Aldrich) at 37°C. The different bacterial suspensions were prepared according to the 0.5 McFarland standard. For this, a stock suspension was prepared at turbidity 0.5 McFarland standard (corresponding to an approximate concentration of 1.5 × 10^8^ colony-forming units (CFU)/mL) from 24-hour cultures on Muller Hinton agar (MHA) and then diluted to 10^6^ CFU/mL for the tests.

#### 2.3.3. Determination of Minimum Inhibitory Concentrations

The inhibition parameter of the extract was assessed by determining the minimum inhibitory concentrations (MICs) using the protocol M07-A9 described by the Clinical and Laboratory Standards Institute (CLSI) in 2012 and Abdoulahi et al. in 2023 [8, 9]. The tests were carried out in triplicates in sterile 96-well microplates. In fact, 160 *μ*L of Muller Hinton broth (Sigma Aldrich) (MHB) culture medium was introduced into the first wells and 100 *μ*L into the rest of the wells. Subsequently, 40 *μ*L of a sterile solution of concentrated extract at 10 mg/mL was taken and introduced into the corresponding wells and followed by a series of 5 serial dilutions of geometry of order 2. Finally, 100 *μ*L of a bacterial suspension with a load of 10^6^ cells/mL was distributed into the test wells and those of the negative control. The concentrations of extracts and ciprofloxacin in the wells ranged from 1000 *μ*g/mL to 31.25 *μ*g/mL and from 1.95 *μ*g/mL to 0.0153 *μ*g/mL, respectively, and the final volume in each well was 200 µL, and the final concentration of DMSO was less than 1% with no effect on bacteria growth. The final inoculum load in each well was 5 × 10^5^ cells/mL. The sterility control consisted solely of the culture medium. Ciprofloxacin is used as positive control. The microplates were incubated at 37°C for 24 hours. At the end of the incubation period, 10 *μ*L of resazurin solution freshly prepared at 0.15 mg/mL was added in all wells and the plates were incubated again in the same conditions for 30 minutes. The lowest concentration at which there was no change of coloration from blue to pink corresponding to the absence of visible bacterial growth was considered as the MIC.

### 2.4. Antioxidant Activity by DPPH^•^

The antioxidant power of the crude extract of *Detarium microcarpum* (DeM) was estimated by comparison with a natural antioxidant (ascorbic acid AnalaR NORMAPHUR). All tests were carried out in three replicates for each concentration. DPPH^·^ radical scavenging activity was measured according to the protocol of Brand-Williams et al., in 1995, where 10 *μ*L of each of the ethanolic DeM solutions tested at different concentrations were mixed with 195 *μ*L of an ethanolic solution of DPPH^•^ (Sigma Aldrich) (120 *µ*M) in a 96-well microplates [11]. After 30 minutes of incubation period in the dark at laboratory temperature, absorbance was read at 517 nm using a spectrophotometer UV-Vis. Inhibition of the DPPH^·^ free radical by ascorbic acid was also analysed for comparison.

#### 2.4.1. Determination of the Percentage of Inhibition

Free radical inhibition in percentage (*I* %) was calculated using the following formula:

(1) $$\begin{array}{c} {\text{DPPH}}^{·}\text{scavenging effects }\left(\%\right)\left[\left(\frac{A_{0}-A_{1}}{A_{0}}\right)\times 100\right], \end{array}$$

*A*_0_ and *A*_1_ correspond to the absorbances at 540 nm of the radical (DPPH^·^) in the absence and presence of antioxidants, respectively.

#### 2.4.2. Estimation of Reaction Kinetics

The reaction kinetics and the parameters for calculating the antioxidant activity for ascorbic acid and the DeM concentrations studied were calculated.

(2) $$\begin{array}{c} \%{\text{DPPH}}^{·}\text{remaining}=\left(\frac{A_{t}}{A_{0}}\right)\times 100, \end{array}$$

where *A*_0_ and *A*_*t*_ correspond to the absorbance at 540 nm of DPPH^·^ at initial and steady states, respectively. *A*_*t*_ value was obtained at the steady state region where absorbance did not depict further observable decreases.

### 2.5. Antioxidant by ABTS Assay

Antioxidant activity was measured in accordance with the method of Rafael et al., in 2004. The ABTS (Sigma Aldrich) solution was prepared at 7 mM by dissolving the ABTS in water and treated with 2.45 mM of potassium persulphate, and the mixture is left to stand at room temperature for 12–16 h. The solution was then diluted with ethanol to give an absorbance of 0.7 at 734 nm. 10 *µ*L of the extract or standard solution are introduced in each corresponding well, and 190 *µ*L of the diluted ABTS solution were added. Ascorbic acid is used as the standard prepared in ethanol (96%) [12].

(3) $$\begin{array}{c} \%\text{scavenging}=\left(\frac{1-\;A_{S}}{A_{0}}\right)\times 100, \end{array}$$

where *A*_0_ is the absorbance without sample and *A*_*S*_ is the absorbance with sample.

### 2.6. H_2_O_2_ Scavenging Capacity Assay

Antioxidant activity was measured in accordance with the method of Wang et al., in 2000. The H_2_O_2_ solution (2 mM) is prepared in a phosphate buffer (pH 7.4) at 50 mM. In the 96 well plate, 18.2 *μ*L of extract at different concentrations and or standard, 72.7 *μ*L of 50 mM phosphate buffer, and then 109 *μ*L of H_2_O_2_ (2 mM) are, respectively, introduced. The absorbance is read at 230 nm after 10 minutes of incubation. 50 mM phosphate buffer without H_2_O_2_ is used as a blank [13]. Hydrogen peroxide scavenging ability (in triplicate) is calculated by the following formula:

(4) $$\begin{array}{c} \%\text{scavenging}=\left(\frac{1-\;A_{S}}{A_{0}}\right)\times 100, \end{array}$$

where *A*_0_ is the absorbance without sample and *A*_*S*_ is the absorbance with sample.

### 2.7. Acute Oral Toxicity Evaluation

2-month-old NMRI mice weighing between 27 and 32 g were used to study the acute oral toxicity. The acute oral toxicity test was conducted using the “dose adjustment” method of OECD line 425 (2008) and involved testing *D. microcarpum* root bark extract at a single dose of 2000 mg/kg body weight [14]. The test was carried out on 6 NMRI mice. They were fasted for 24 hours and then divided as follows: control lot consisting of 3 NMRI mice receiving a normal saline solution (NaCl 9‰) at a dose of 5 mL/kg and experimental lot consisting of 3 mice receiving the extract, at a dose of 2000 mg/kg. Behavioural monitoring was performed for 6 h after administration of the extract. The mice were then fed and hydrated ad libitum. At D0, D1, D7, and D14, the mice were weighed and sampled for blood analysis. Liver (alanine aminotransferase (ALAT) and aspartate aminotransferase (ASAT)), kidney (creatine and creatininemia), and immune (red and white blood cell counts) organ balances were assessed.

### 2.8. Statistical Analysis

Data were expressed as the mean ± standard deviation (m ± S.D). One-way analysis of variance (ANOVA) followed by Dunnett's test was used to determine the degree of statistical significance of results. Difference was considered as significant at *p* < 0.05.

## 3. Results

### 3.1. Extraction Yield

The choice of this plant was based on the literature on ethnobotany in Niger, and the results of previous surveys carried out by several researchers were used. Five solvents were used to prepare five organic extracts, with extraction yields varying from 13.1 to 34.7 percent (Table 1). The extraction yields were dependent on the extraction solvent.

### 3.2. Anti-*Shigella* Activity of *D. microcarpum* Root Bark

The minimum inhibitory concentrations of *D. microcarpum* root extracts vary from 500 to 1000 µg/mL on *shigella* subhasis (Table 2).

### 3.3. Antioxidant Activity of the Methanolic Crude Extract

#### 3.3.1. Estimation of the Kinetics of the Reaction

**Figure 1:**
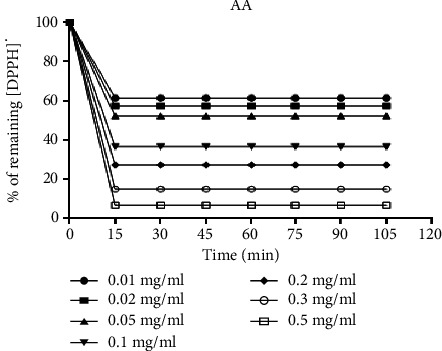
The kinetics of reduction of the DPPH^·^ free radical with the ascorbic acid (AA).

**Figure 2:**
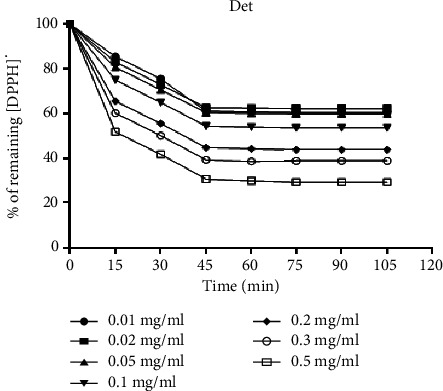
The kinetics of reduction of the DPPH· free radical with the methanolic extract of *D. microcarpum* (Det).

The kinetics of reduction of the DPPH^·^ free radical obtained are shown for each concentration of ascorbic acid in Figure 1 and in Figure 2 for the methanolic extract of *D. microcarpum*.

Table 3 shows the antioxidant activity of the methanolic extract of *D. microcarpum*.

### 3.4. Acute Oral Toxicity of *D. microcarpum* Root Bark

Table 4 shows the results of oral acute toxicity.

## 4. Discussion

### 4.1. Extraction Yield

The extraction yield expressed as a percentage of nonvolatile compounds is shown in Table 1. Analysis of this table shows that the water-ethanol extract (34.7%) from the root bark has the highest yield, followed by the methanol extract (27.5%). The lowest yield was obtained for the dichloromethane extract (10.2%). These results are important for assay analysis to quantify total secondary metabolism levels and evaluate the various biological assays.

### 4.2. Anti-*Shigella* Activity of *D. microcarpum* Root Bark

Among the 4 bacterial strains tested, two were susceptibles, *Shigella boydii* and *Shigella flexneri* (Table 2). According to the criteria of Kuete in 2010 [10] (Table 5), the dichloromethane crude extract was inactive against these two strains (*Shigella boydii* and *Shigella flexneri*). The crude methanol, ethanol, and water/ethanol extracts showed the best activity with MIC at 500 *µ*g/mL against the *Shigella boydii* strain. On *Shigella flexneri* only the methanolic extract showed moderate activity at 1000 *µ*g/mL.

Previous phytochemical studies of the methanolic extract of the *D. microcarpum* root bark revealed the presence of sterols and polyterpenes, saponins, and polyphenols such as gallic and catechic tannins, flavonoids [15], antioxidant activity [16], and anthelmintic activity [17]. Polyphenols help stop haemorrhaging and fight microbial infections. Plants rich in polyphenols are used to tighten supple tissues, as in the case of venous veins, to drain excessive secretions, as in diarrhoea, and to repair tissues damaged by burns [18].

Bioguided fractionation of this methanolic extract of *D. microcarpum* could make it possible to precisely isolate the compounds responsible for the anti-*shigella* activity. However, it would appear that this activity is due to the joint, possibly synergistic, action of all these classes of compounds.

In addition, the reference antibiotic (ciprofloxacin) showed greater anti-*shigella* activity than the plant extract tested. The reference antibiotics are pure molecules [19], whereas all the extracts are nonpurified mixtures of substances. Plant extract is a mixture of many types of molecules that can have synergistic effect or antagonist.

### 4.3. Antioxidant Activities

Concerning the kinetics of reduction of the DPPH^·^ free radical, the reaction is biphasic, with a rapid drop in absorbance in the first few minutes, followed by a slower stage until equilibrium is reached, for the concentrations tested for the crude extract and the positive control both (Figures 1 and 2), two zones can be distinguished.

The first one is the zone of high radical trapping kinetics observed after the first 15 minutes for ascorbic acid and Det and the zone of low DPPH^·^ radical trapping kinetics or zone of tendency towards equilibrium observed after 15 minutes for ascorbic acid and after 45 minutes for Det. The results show that the reaction between DPPH^·^ and ascorbic acid, Det, reaches equilibrium after a short time compared with Det.

For the antioxidant activity results (Table 3), it appears that the methanolic crude extract of *D. microcarpum* shows good antioxidant activity with the radicals DPPH^·^ and ABTS with IC_50_ at 228 and 191 *µ*g/mL, respectively. The activity on H_2_O_2_ is lower than the others where the IC_50_ is at 233 *µ*g/mL. Based on the statistical analysis, there was a significant difference between the antioxidant activity of the methanolic extract of *D. microcarpum* and that and ascorbic acid. This antioxidant activity could be linked to the chemical composition of this plant, and previously, some authors showed that this plant contained polyphenolic compounds, tannins, and flavonoids that are responsible for antioxidant activity [15, 16]. These results could justify the antibacterial activity of this extract.

### 4.4. Oral Acute Toxicity

Family self-medication is based on ancestral knowledge and the knowledge of traditional practitioners and herbalists about medicinal plants [20]. The recipes prescribed by herbalists and traditional practitioners have quality standards at all, as there are no formulations for traditional recipes from Niger. In addition, the risks of toxicity due to unfamiliarity with plants and the lack of a defined dosage and appropriate instructions for use mean that people need to be aware of the dangers of taking herbal medicines.

### 4.5. Behavioural Parameters

Oral administration of a single dose of 2000 mg/kg body weight of *Detarium microcarpum* root extract did not cause any deaths in the mice treated during the 14th day of experimentation. However, a number of changes were observed immediately after administration of the extract, such as restlessness, a decrease in mobility, and an increase in drowsiness during the first two days, but they returned to normal after the third day.

No particular signs of acute oral toxicity were observed in mice for 14 days. The LD_50_ was estimated to be greater than 2000 mg/kg, suggesting that *Detarium microcarpum* root macerate is safe to use.

### 4.6. Liver, Kidney, and Haematological Parameters

Transaminases or amino transferases are tissue enzymes that catalyse the transfer of alpha-amino radicals from alanine and aspartic acid to alpha-ketoglutaric acid.

ALAT transaminases are present in the liver, but also in muscle, and are more specific for liver and can help know an eventual damage of this organ [21, 22]. ALAT is a cytosolic enzyme secreted by liver cells from which it is released into the blood in the hepatic cell necrosis [23]. ASAT, found in the kidneys, pancreas, lungs, and skeletal muscle, is an indicator of hepatocyte destruction and is slightly more sensitive [21].

ALAT and ASAT levels rise rapidly when the liver is damaged for many reasons, including hepatic cell necrosis, hepatitis, cirrhosis, and the hepatotoxicity of certain drugs [22, 23].

In this study, the concentration of these two enzymes (ALAT and ASAT) was significantly reduced in animals treated with a single dose of 2000 mg/kg body weight (Table 4), suggesting that the extract has a hepatoprotective action at this dose. The statistical analysis shows that ALAT values vary significantly between D0, D1, and D7; There was a nonsignificant difference in ASAT value between D0 and D1; the difference was significant between D1, D7, and D14, suggesting that the extract has a hepatoprotective action at this dose.

The analysis of renal functions (uraemia and creatininaemia) revealed that administration of the extract did not show significant changes between D0 and D7 and the difference was significant, the D14 for creatininaemia. Serum uraemia and creatininaemia are considered the main markers of nephrotoxicity [24].

Haematological analysis (red and white blood cells) revealed no significant changes in mice treated with the single dose of 2000 mg/kg for white cells between D0 and D14, and the difference was significant, the D14 for red cells (Table 4).

### 4.7. Chemical Composition of *D. microcarpum* Extract

Considering the medicinal importance of this tree in West Africa, several phytochemical and pharmacological studies were conducted on the different organs of *D. microcarpum.*

Phytochemical screening showed that the methanolic extract of *D. microcarpum* root bark contains sterols and polyterpenes, polyphenols, flavonoids, tannins, saponosides, anthocyanins, and coumarins [15, 25].

The methanol extract of *D. microcarpum* roots and its fraction significantly reduced blood glucose levels in alloxan-diabetic rats without producing hypoglycemia, an effect attributed to the flavonoids abundantly present in the extract [26].

Previous studies have revealed that the methanol extract and its fractions of *D. microcarpum* root bark produced 100% lethality on ground glass (*pheretima posthuma*) at the concentration of 2 mg/mL after a time (1–3 h), compared with the results of other researchers working on other ground glass species and other plants (lethality time greater than 6 h) [27–30]. The doses of the extract used to obtain the 100% lethality time would explain the differences observed.

Chemical profiles carried out on the methanolic extract revealed the presence of flavonoids and tannins [15], while studies by Vidyadhar et al., 2010, and Deore et al., 2009, showed that flavonoids and tannins are involved in antiparasitic and antibacterial activity.

Rhinocerotinoic acid has been isolated from root barks and shows activities against *Salmonella typhi* and *Salmonella enteritidis* [31].

## 5. Conclusion

This work shows that ethanolic, methanolic, and hydroethanolic extracts of *D. microcarpum* root bark are the best active against the *shigella flexneri* strain. This result demonstrates the anti-*shigella* potential of root barks and contributes to the justification of using this plant in the treatment of gastrointestinal disorders. The methanolic crude extract of *D. microcarpum* has a good antioxidant activity. The results of *in vivo* oral toxicity tests on mice showed no acute toxicity for a dose less than or equal to 2000 mg/kg for 14 days. The LD_50_ was estimated to be greater than 2000 mg/kg, which suggests that *D. microcarpum* root macerate is safe to use. Further studies are needed to perform a bioguided isolation of active compounds against *shigella*.

## Figures and Tables

**Table 1:** Extraction yield.

Solvent	MeOH	EtOH	CH_2_Cl_2_	Water-EtOH (30/70)	MeOH-CH_2_Cl_2_ (1/1)
Yield (%)	27.5	26.8	10.2	34.7	13.1

**Table 2:** Minimum inhibitory concentrations of extracts.

MIC (*µ*g/mL)				
Bacterial strains	SB NR 521	SO NR 519	SF NR 518	SD CPC
EDM	**500**	>1000	>1000	>1000
DMDM	**1000**	>1000	>1000	>1000
DDM	>1000	>1000	>1000	>1000
EWDM	**500**	>1000	>1000	>1000
MDM	500	>1000	1000	>1000
CP	0.015	0.030	0.030	0.060

SB NR 521: *Shigella boydii*, SO NR 519: *Shigella sonnei*, SF NR 518: *Shigella flexneri*, SD CPC: *Shigella dysenteria*, EDM: ethanol crude extract, DMDM: dichloromethane-methanol crude extract (1 : 1), DDM: dichloromethane crude extract, EWDM: water-ethanol crude extract (7 : 3), MDM: methanol crude extract, CP: ciprofloxacin, CPC: Centre Pasteur du Cameroun. The values in bold show the most active extracts.

**Table 3:** Antioxidant activity of methanolic extract of *D. microcarpum*.

Plants/control	Extracts/reference	DPPH^·^ IC_50_ *µ*g/mL	ABTS IC_50_ *µ*g/mL	H_2_O_2_ IC_50_ *µ*g/mL
*D. microcarpum*	Methanolic	228 ± 0.04^∗∗∗^	191 ± 0.2^∗∗∗^	233 ± 0.07^∗∗∗^

Positive control	Ascorbic acid	7.36 ± 0.31	19 ± 0.03	19.1 ± 0.02
	Quercetin	21 ± 0.02	NT	NT

NT: not tested; (^∗∗∗^) = *p* < 0.001.

**Table 4:** Effects of methanolic extract on liver, kidney, and haematological parameters.

Parameters analysed	D0	D1	D7	D14
ALAT (UI/L)	49.13 ± 0.14	47.17 ± 0.34^∗∗^	44.23 ± 01.35^∗^	41.00 ± 3.03^ns^
ASAT (UI/L)	102.30 ± 10.42	101.90 ± 7.14^ns^	99.40 ± 2.10^∗^	96.26 ± 6.00^∗∗^
Uraemia (g/L)	0.53 ± 0.01	0.53 ± 0.03^ns^	0.58 ± 0.00^∗^	0.60 ± 0.06^ns^
Creatininaemia (mg/L)	9 ± 2	10 ± 1^ns^	9 ± 3^ns^	8 ± 2^∗∗^
Red blood cells (10^6^/*µ*L)	9.66 ± 0.17	8.73 ± 0.44^ns^	9.30 ± 0.55^ns^	9.54 ± 0.49^∗∗^
White blood cells (10^3^/*µ*L)	13.06 ± 3.25	11.57 ± 2.10^ns^	14.84 ± 5.31^ns^	10.51 ± 4.11^ns^

ALAT: alanine amino-transférase; ASAT: aspartate amino-transférase, (^∗^) = *p* < 0.033; (^∗∗^) = *p* < 0.002; ns = not significant.

**Table 5:** Scale for assessing the anti-*shigella* activity of extracts as a function of MIC values [10].

Extract	Activity
CMI < 100 *μ*g/mL	Excellent
100 < CMI < 512 *μ*g/mL	Strong
512 < CMI < 2048 *μ*g/mL	Moderate
CMI > 2048 *μ*g/mL	Low
CMI > 10 mg/mL	Inactive

## Data Availability

The data used to support the findings of this study are available from the corresponding author upon request.
